# Functional, pathogenic, and pharmacological roles of protein folding intermediates

**DOI:** 10.1002/prot.26479

**Published:** 2023-02-27

**Authors:** Emiliano Biasini, Pietro Faccioli

**Affiliations:** ^1^ Department of Cellular Computational and Integrative Biology (CIBIO) University of Trento Trento Italy; ^2^ Department of Physics University of Trento Trento Italy; ^3^ Trento Institute for Fundamental Physics and Applications Italian Institute for Nuclear Physics Trento Italy

**Keywords:** drug discovery, misfolding, molecular simulations, protein folding, protein homeostasis

## Abstract

Protein expression and function in eukaryotic cells are tightly harmonized processes modulated by the combination of different layers of regulation, including transcription, processing, stability, and translation of messenger RNA, as well as assembly, maturation, sorting, recycling, and degradation of polypeptides. Integrating all these pathways and the protein quality control machinery, deputed to avoid the production and accumulation of aberrantly folded proteins, determines protein homeostasis. Over the last decade, the combined development of accurate time‐resolved experimental techniques and efficient computer simulations has opened the possibility of investigating biological mechanisms at atomic resolution with physics‐based models. A meaningful example is the reconstruction of protein folding pathways at atomic resolution, which has enabled the characterization of the folding kinetics of biologically relevant globular proteins consisting of a few hundred amino acids. Combining these innovative computational technologies with rigorous experimental approaches reveals the existence of non‐native metastable states transiently appearing along the folding process of such proteins. Here, we review the primary evidence indicating that these protein folding intermediates could play roles in disparate biological processes, from the posttranslational regulation of protein expression to disease‐relevant protein misfolding mechanisms. Finally, we discuss how the information encoded into protein folding pathways could be exploited to design an entirely new generation of pharmacological agents capable of promoting the selective degradation of protein targets.

## 1 INTRODUCTION

In its classical definition, the native state of a protein refers to the three‐dimensional conformation of the polypeptide that determines its biological activity. Such a conformation is reached at the end of a complex series of structural transitions that each chain of amino acids undergoes soon after exiting the ribosomal channel, collectively referred to as the protein folding process. A correct folding pathway for newly synthesized proteins is fundamental for maintaining biological activities, influencing the rate and efficiency of protein expression and functions. This process is, in fact, closely monitored by multiple cellular mechanisms, which intervene to correct aberrantly folded species or to reroute them to degradation in the proteasome or the lysosomes. These mechanisms collectively guarantee protein homeostasis, also called proteostasis.

Theoretical and experimental evidence gathered over the last 30 years have established that the thermodynamics and kinetics of the folding of single‐domain globular proteins are determined by the polypeptide chain's multidimensional free‐energy landscape, which is funneled toward the native state.^1^ The critical hypothesis underlying the so‐called energy landscape theory is that natural evolution has selected sequences of amino acids that minimize the intrinsic frustration associated with ordinary heteropolymers. The result is a highly cooperative transition that enables the chain to effectively reach its minimum energy structure by avoiding the random exploration of the whole space of configurations. In the extreme limit of a completely smooth energy landscape, proteins can roll down their folding funnel from any unfolded configuration and reach the native state via virtually infinite different folding pathways. An important prediction of this idealized model is that the folding transition should obey simple two‐state kinetics, a feature confirmed by physics‐based microscopic simulations^2^ and by experiments carried out on small globular proteins.^3^

In contrast, understanding the folding of larger or multi‐domain proteins has proven much more challenging, both from theoretical and experimental points of view. In fact, the relevant time scales of protein folding can extend to seconds, minutes, or even longer. Moreover, the folding kinetics is often characterized by the population of partially folded intermediates,^4^ which can be prone to misfolding and aggregation.^5^, ^6^ The emerging picture is one in which the energy landscape of biologically relevant proteins is much more rugged than that of small globular proteins. This feature enables the emergence of predominant folding pathways. In particular, the folding of large proteins seems to occur through the rapid and cooperative formation of a few small structural building blocks (foldons), which then dock together to form the structure.^7^ All these concepts imply that the landscape of conformations that a protein may explore during the folding process includes a variety of dynamically interconverting metastable species, different from the native state, whose abundance and thermodynamics principles govern kinetics. Such a perspective suggests that if the docking of foldons occurs through relatively consistent steps, intermediate conformations appearing along the process could play fundamental roles by affecting both the rate and efficiency of protein folding. While the relevance of the whole folding process in modulating proteostasis is conceptually established, there is so far insufficient evidence that non‐native protein states appearing along the folding pathways might hold relevant biological information and be directly implicated in functional or regulatory events.

In the last three decades, computer simulations have played a growing role in establishing a physics‐rooted understanding of protein folding. In particular, molecular dynamics (MD) simulations can provide an accurate atomically detailed representation of the folding transition, thus elucidating the energy landscape's structure and identifying the prominent folding mechanisms. Unfortunately, even relying on the most powerful special‐purpose supercomputer, all‐atom MD simulations are extremely computationally expensive and can only be used to study the folding of small polypeptide chains with folding times up to the millisecond scale.^2^ Thus, while plain MD simulations can offer valuable insights into the folding kinetics of very small chains or the cooperative formation of protein domains or foldons, they do not provide a viable tool to investigate the kinetics involving the interconversion between partially folded structures. The development of computational enhanced‐sampling techniques has been partially able to overcome those limitations, allowing for interesting mechanistic insights into the folding process of biologically relevant proteins.^8^

The combination of these innovative computational technologies with rigorous experimental approaches has recently provided evidence indicating that protein folding intermediates could play roles in disparate physiological and pathological processes. We refer to these protein conformers as functional protein folding intermediates to show precise protein structures appearing along the folding process and distinct from the native state that serves a specific function in the cell. Importantly, these intermediates are not considered misfolded but rather transiently populated in a dynamic equilibrium with the native state. This manuscript will review relevant literature regarding functional protein folding intermediates and their involvement in the posttranslational regulation of protein expression and protein misfolding disorders. We will also discuss new and unexpected pharmacological paradigms arising from the knowledge of functional protein folding intermediates and their unique biology.

The manuscript is organized as follows. In Section 2, we briefly review some enhanced sampling schemes and approximations that have enabled, for the first time, the all‐atom simulations of large proteins and the structural characterization of folding intermediates. Sections 2 and 3 discuss specific study cases, highlighting how folding intermediates can play an essential role in protein function, (mis‐)regulation, and disease‐relevant aggregation. Section 4 presents the hypothesis that folding intermediates can provide an unappreciated role in regulating protein homeostasis via posttranslational modifications (PTMs). In Section 5, we discuss how these studies have led to the development of an entirely new paradigm for drug discovery based on reducing the expression level of proteins by targeting folding intermediates. Conclusions and outlooks are summarized in Section 6.

## 2 SIMULATIONS OF PROTEIN FOLDING, MISFOLDING, AND AGGREGATION WITH ALL‐ATOM PHYSICS‐BASED MODELS

All‐atom MD simulations can aptly complement experiments investigating the kinetics of complex protein transitions, including in principle folding, misfolding and aggregation. The advantage of MD simulations is that they rely on physics‐based theoretical modeling to yield the atomically resolved reconstruction of structural rearrangements. Unfortunately, in practice, even depending on the most powerful special‐purpose supercomputer (Anton, developed by D.E.S. Research), MD simulations can only cover time intervals of up to a few milliseconds for polypeptide chains consisting of nearly 100 amino acids,^2^ which are considerably smaller than most proteins of biological interest. While the next generations of the Anton supercomputer may be able to increase the time windows accessible to MD, it is unlikely that a brute‐force MD scheme will enable the simulation of many critical biomolecular processes that can occur at the time scales of seconds or longer and can involve several hundreds of amino acids.

Given the computational limitations of MD, more sophisticated alternative algorithms have been developed and then applied to investigate rare biological transitions.^9^ Most of these schemes typically require complementing MD simulations with additional information, such as identifying the slowest collective variables (CVs) in the system or choosing a good reaction coordinate. To the best of our knowledge, to date, the only enhanced sampling technique that has been systematically applied to perform all‐atom simulations of the folding, misfolding, and aggregation of proteins consisting of several hundreds of amino acids are the so‐called bias functional (BF) approach^10^ and its evolution called self‐consistent path sampling (SCPS).^11^ In the BF scheme, a specific type of biased dynamics called ratchet‐and‐pawl MD (rMD) is employed to efficiently generate a statistically significant number of productive trajectories. In rMD, an auxiliary history‐dependent potential depending on a suitable CV is introduced to prevent the chain from backtracking toward the initial state.^12^ Conversely, the biasing force is inactive when the system spontaneously progresses toward the final state. It has been shown that, in the ideal case in which the CV is an ideal reaction coordinate (the so‐called committor function), rMD simulations yield the correct Boltzmann sampling in the region explored by the transition path ensemble.^13^, ^14^ On the other hand, in practical applications, rMD must rely on a proxy of the ideal reaction coordinate, so it yields only an approximate sampling of the equilibrium distribution. The BF approach provides a scheme to keep the systematic errors of rMD to a minimum. In this approach, a variational principle derived from Langevin dynamics is used for scoring the reactive trajectories generated by rMD to identify those with the highest probability of occurring in the absence of any biasing force.^10^

SCPS exploits a different strategy to mitigate the systematic errors of plain rMD simulations: It is based on an iterative procedure through which the reactive rMD trajectories obtained in the previous iteration are analyzed to obtain a better proxy of the reaction coordinate, which is then used in the next iteration of rMD to generate improved reactive trajectories. It has been shown that the folding mechanism obtained by BF and SCPS schemes is statistically indistinguishable from that predicted by the plain MD simulations performed on the Anton Supercomputer.^11^ The same enhanced sampling schemes have also been extensively validated against the result of different biophysical experiments.^15^, ^16^, ^17^ SCPS and BF have then been applied to several case studies of biological interest to be discussed below, to yield a complete structural characterization of protein folding intermediates.

## 3 FUNCTIONAL ROLE OF FOLDING INTERMEDIATE IN PROTEIN AGGREGATION, FUNCTION, AND REGULATION, CASE I: PRIONS

Proteins evolve under two kinds of evolutionary pressure, one selecting their biological function, the other challenging their ability to remain soluble under physiological conditions. However, in several pathologies, specific proteins lose their native fold and acquire a different conformation, ultimately clustering into aberrant aggregates.^18^ The phenomenon, known as protein misfolding, lies at the root of a wide variety of human diseases, such as neurodegenerative disorders, in which protein aggregation occurs in the brain. Prion diseases, also known as transmissible spongiform encephalopathies, represent a meaningful example. These disorders have the peculiarity of manifesting in a sporadic, inherited, or transmissible fashion. All forms of prion diseases, including Creutzfeldt–Jakob disease (CJD), fatal familial insomnia, and Gerstmann–Sträussler–Scheinker syndrome, are associated with the conformational conversion of the cellular prion protein (PrP), a cell surface glycoprotein mainly expressed in the central nervous system, into a misfolded isoform (PrP^Sc^) that accumulates in the brain of affected individuals.^19^ PrP^Sc^ is a proteinaceous infectious particle (prion) capable of multiplying by directly recruiting other PrP molecules and causing their conformational rearrangement into growing PrP^Sc^ polymers.^20^, ^21^ Despite the recent elucidation by cryogenic electron microscopy of the structure of PrP^Sc^ as a parallel‐in‐register‐β‐sheet fiber^22^, ^23^ and decades of research from multiple laboratories, the definition of the mechanism underlying prion replication at atomic resolution has proven to be an unprecedented experimental challenge, mainly due to the lack of techniques combining deep spatial and temporal resolution.^24^

Previous results collected using stopped‐flow and force spectroscopy techniques suggested that a relatively stable folding intermediate is present along the folding pathway of PrP.^25^, ^26^, ^27^ However, they failed to provide high‐resolution structural information about it. Interestingly, three more recent studies further supported the existence of a PrP folding intermediate. In the first one,^28^ an intermediate folding conformer of a disease‐relevant PrP mutant (T183A) was identified using NMR spectroscopy, biophysics, and metadynamics simulations. Strikingly, this conformer was nearly identical to a PrP folding intermediate identified in coarse‐grained^29^ and in all‐atom simulations,^30^ the latter based on the BF approach described in Section 2. Interestingly, computational and biochemical experiments revealed that such a PrP conformer might also spontaneously appear during the structural fluctuation of the protein starting from its native state, implying that its appearance in a cell may not be strictly limited to the subcellular compartment where the protein folds (i.e., the endoplasmic reticulum) but also occur on the cell surface, where the mature protein typically resides.^28^, ^30^ Collectively, these data suggest that in addition to its role in the normal folding pathway of the protein, a folding intermediate could be involved in other biological aspects of PrP, including prion conversion, by acting as a direct substrate for PrP^Sc^ replication.

## 4 FUNCTIONAL ROLE OF FOLDING INTERMEDIATE IN PROTEIN AGGREGATION, FUNCTION AND REGULATION, CASE II: SERPINS

Serpins (serine protease inhibitors) provide another notable example of proteins that are associated with a family of misfolding pathologies (serpinopathies) that are induced mainly by the pathogenic dysregulation of their target proteases.^31^ The most common serpinopathies are induced by mutations in the canonical secretory serpin a1‐antitrypsin (A1AT) responsible for regulating leukocyte serine proteases. For example, the relatively benign S‐mutation (Glu264Val) promotes the population of a state of misfolded conformations structurally and kinetically close to the native state. The resulting lung diseases can be slowed (although not wholly halted) by A1AT augmentation therapy.^32^ On the other hand, the highly pathogenic Z‐mutation (Glu342Lys) is associated with an irreversible and aggregation‐prone misfolded conformation. The resulting shallow levels of circulating active A1AT lead to severe liver diseases, for which the only known effective treatment is liver transplants.^33^

**FIGURE 1:**
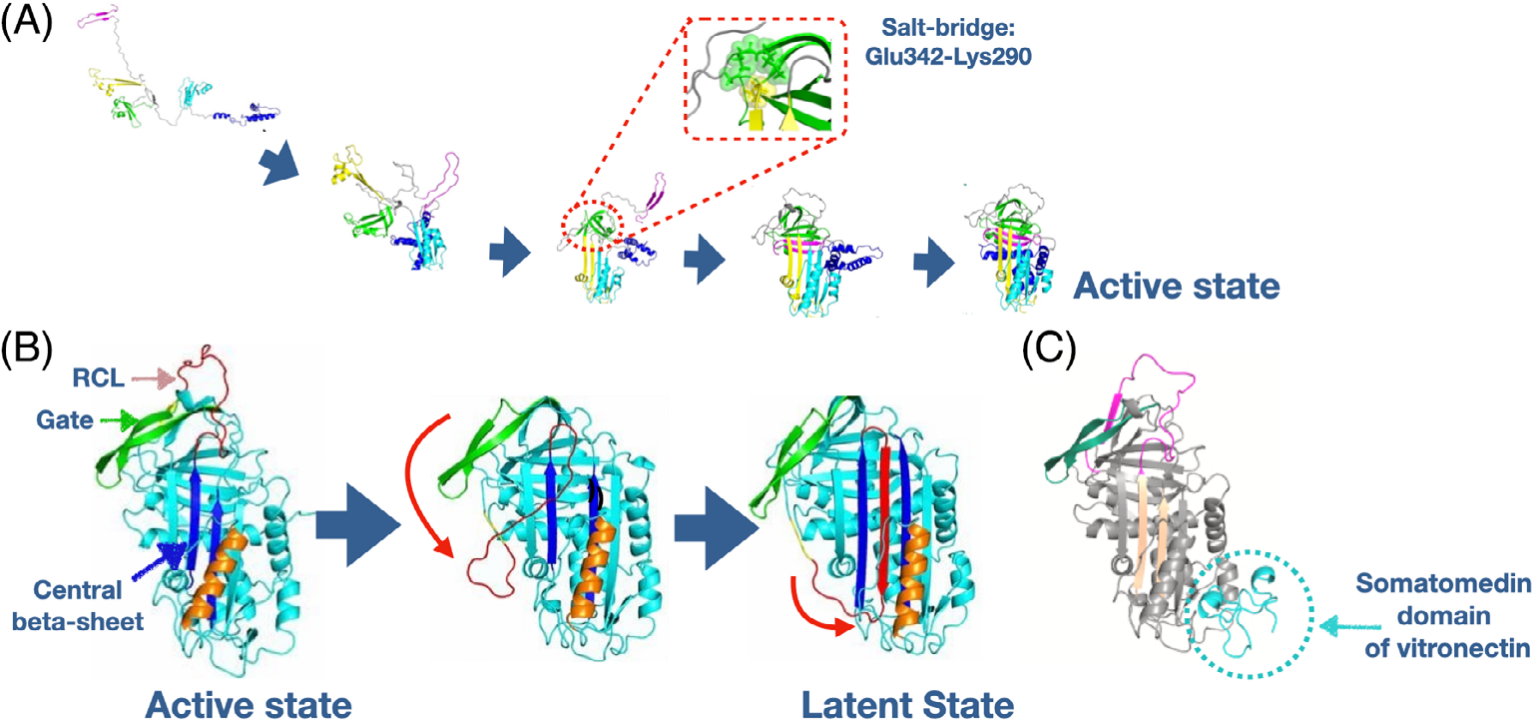
All‐atom simulations serpin folding and latency transition. Panel (A): Schematic representation of a typical folding pathway for A1AT serpin observed in bias functional (BF) simulation. The red‐dotted circle highlights the formation of the salt‐bridge that is affected by the Z‐ and S‐ mutation. Panel (B): Schematic representation of the spontaneous latency transition of plasminogen activator 1 (PAI‐1) serpin, displaying the existence of a meta‐stable pre‐latent intermediate. (C) The binding of Vitronectin to PAI‐1 slows the latency transition by allosterically rigidifying the reactive center loop (RCL) and gate mobility.

Using the BF algorithm described in Section 2, it has become possible to simulate many folding and misfolding events with realistic all‐atom models for different A1AT variants, including the wild‐type, the S‐mutant, and the Z‐mutant.^34^ These theoretical predictions were first validated using the available biophysical experiments to refold wild‐type serpins and later applied to elucidate the chemical–physical mechanism responsible for the misfolding of *S* and *Z* mutants. In agreement with experimental observation, the Z mutation was found in simulations to disrupt the folding efficiency. In particular, an in‐depth analysis of the folding trajectories revealed that the detouring from the correct folding pathway occurs at a relatively early stage of the transition (Figure 1A). A1AT misfolding is driven by the fact that the Z mutation affects an electrostatic interaction between two residues, changing it from attractive (Glu‐Lys) into repulsive (Lys‐Lys). This repulsion prevents the two residues from coming in contact at an early folding stage. If this contact does not occur early in the reaction, the chain cannot reach the native conformation. On the other hand, in the BF simulations for the S‐mutant, a much more significant fraction of successful folding events was observed. The predicted misfolded conformations were much more structurally close to the native state and less aggregation‐prone. The comparative analysis of the successful and unsuccessful folding trajectories revealed that misfolding is associated with an alteration of the same electrostatic interaction involved in the misfolding of the Z‐mutant. However, the S mutation does not turn the attractive Glu‐Lys interaction into a repulsive one (which in Z‐mutant is Lys‐Lys) because the charged residue (Glu) is replaced by a neutral one (Val), so these two residues are not as strongly impeded to come close at the beginning of the folding process. As a result, the detour from the correct folding pathway occurs much more sporadically and at a later stage of the transition. The physicochemical mechanism underpinning the misfolding of A1AT mutants was validated by comparing experiments and simulations of several A1AT variants characterized by suitable rescue mutations. For example, the disruptive effect of the *Z* mutation could be rescued by replacing the Lys residue involved in the repulsive electrostatic interaction with a Glu. This way, the Glu‐Lys pair is turned into a Lys‐Glu, and the attractive electrostatic interaction is preserved. Proteins with such double mutation were predicted and observed to have a folding efficiency comparable to that of the wild‐type.

Another exciting feature of serpin proteins is that their physiological function and regulation rely on meta‐stable chain conformations. Indeed, the interaction with the target protease typically occurs in the so‐called reactive center loop (RCL) and induces a large “mouse‐trap” conformational transition to an inactive stable state, in which the RCL is inserted in a central beta‐sheet (Figure 1B). To reach the central beta‐sheet, the RCL has to overcome an exposed beta‐sheet referred to as the gate. After this irreversible conformational change, the serpin becomes biologically inert. In most cases, the latency transition is initiated by the cleavage of the RCL, which releases a significant amount of mechanical energy. However, in some members of the serpin family, most notably plasminogen activator 1 serpin (PAI‐1), the transition can also occur spontaneously, albeit on a much longer time scale (in the range of hours). Hence, from a kinetic and thermodynamic point of view, the active state of PA1‐1 may be regarded as a folding intermediate.

PAI‐1 negatively regulates blood clot clearance by mechanically inhibiting several serine proteases, including tissue‐type plasminogen activator and urokinase‐type plasminogen activator.^35^ The spontaneous latency transition, therefore, provides a facile way to regulate PAI‐1 activity. In physiological conditions, it is achieved by binding to the cell adhesion factor vitronectin, which leads to a significant increase in the active state half‐life. Since high levels of active PAI‐1 are associated both with poor cancer prognosis and cardiovascular diseases, PAI‐1 inhibitors that accelerate the latency transition are under development.^7^, ^35^, ^36^ For example, incubation of active PAI‐1 with the antibody H4B3 has been shown to accelerate the latency transition in a concentration‐dependent manner. Unfortunately, the lack of detailed molecular mechanisms for PAI‐1 conformational changes has limited drug design efforts. For example, it has been noted that the epitope recognized by H4B3 is not accessible in PAI‐1 active state, thus raising the question of how this antibody can accelerate the transition. Simulations performed in a native‐centric coarse‐grained model have confirmed that direct folding to the latent structure is disfavored by the presence of a large, entropy‐dominated free‐energy barrier.^37^ On the other hand, simulations performed using the BF scheme have made it possible for the first time to obtain a fully atomistic reconstruction of the PAI‐1 latency transition and provide a viable Physicochemical mechanism of action of H4B3.^38^ Indeed, these simulations showed that the latency transition involves an on‐pathway long‐lived intermediate state in which the RCL is only partially inserted in the central loop (Figure 1C), where the interaction with H4B3 can take place. This binding prevents the protein from backtracking to the active state, thus promoting the latency transition's reaction rate.

Another puzzling feature of the PAI‐1 latency transition concerns how the interaction with the cell adhesion factor vitronectin can slow the latency transition. This question was recently addressed by comparing BF simulations of the latency simulation with and without vitronectin.^39^ An analysis of the productive trajectories revealed that vitronectin binding reduces RCL and gate mobility by allosterically rigidifying structural elements over 40 Å away from the binding site, thus blocking the transition to the latent conformation. The same simulations also revealed a cryptic drug‐binding area (identified by crystallography) populated only in the vitronectin‐bound state.

## 5 FUNCTIONAL FOLDING INTERMEDIATES AND PTMs

PTMs are chemical changes that may occur to a protein after it is translated from its corresponding RNA molecule. These modifications include adding or removing chemical groups or entire molecules by specific enzymes and can occur on the protein's amino acid side chains or backbone. Known types of PTMs include, among others, proteolytic cleavage, glycosylation, phosphorylation, acetylation, methylation, sumoylation, and ubiquitination. PTMs can have a wide range of effects on a protein's structure, function, and stability. For example, adding a phosphate group can change a protein's activity or strength, while adding a ubiquitin moiety may reroute the protein to degradation. Notably, the specific combination of PTMs on a given protein is highly regulated and can change in response to different cellular stimuli. Overall, PTMs play a critical role in regulating protein activity, stability, and localization and can also significantly impact a wide range of cellular processes, such as signaling pathways, enzyme activity, and protein–protein interactions.

PTMs are generally thought to occur on native states of proteins. However, PTMs could theoretically also target long‐lived protein folding intermediates. There is ample evidence, for example, that glycosyl‐ and glycosyl‐phosphatidylinositol chains occur co‐translationally as soon as a polypeptide starts exiting the ribosomal channel.^40^ In principle, any PTM targeting a protein folding intermediate could alter the folding pathway, thus impacting the expression rate of the protein. Regulating the homeostasis of protein folding intermediates, rather than just acting on transcription, translation, or clearance of mature protein forms, could theoretically be energetically favorable and allow the cell to respond rapidly to stimuli. Interesting evidence supporting this hypothesis has recently emerged from the analysis of the mtcPTM database, which contains information about phosphorylation sites identified through mass spectrometry‐based experiments.^41^ A significant proportion (~15%) of all known phosphorylation sites exhibits solvent accessibility scores of their side chains lower than 10% in the unmodified form of the protein and under physiological conditions. Such a high proportion of hidden phosphosites is striking and unexpected, as an inaccessible positioning would not only dramatically hamper the ability of kinases to reach their target sites but also generate an energetically unfavorable environment for hosting the steric and electrostatic properties of a phosphate group. A plausible explanation for such observation is that the target structures of these phosphorylation events on hidden residues are non‐native, functional folding intermediate conformers.

Direct evidence for a functional role of protein folding intermediates in PTM‐mediated regulation of protein expression emerged from the analysis of the regulatory mechanism of the androgen receptor (AR), a paradigmatic example of proteins undergoing folding‐upon‐binding transition. This AR is responsible for the hormone‐dependent transcription of genes involved in cell proliferation and differentiation. Once produced in the Leydig cells of the testes, testosterone circulates in the bloodstream bound to serum sex hormone‐binding globulin. The free form of this hormone can cross the cell membrane and may be converted into 5‐dihydrotestosterone (DHT) in the cytoplasm. The AR's inactive form resides in the cytoplasm bound to heat shock proteins, which are then displaced upon binding of the AR to DHT, leading the receptor to reach its functional conformation, translocate into the nucleus, and activate transcription of target genes. Ligand binding to the AR stabilizes its conformation by inducing an intramolecular structural reorganization of helix 12 that caps the ligand‐binding pocket.

Additionally, the AR could be regulated by several PTMs, including phosphorylation, ubiquitination, SUMOylation, acetylation, and methylation. In one of these cases, it has been shown that serine 791 (S791) within the ligand‐binding domain of the AR could be phosphorylated by protein kinase B. This modification inhibits ligand interaction and promotes polyubiquitination and degradation of the AR by the proteasome. Interestingly, in natively folded, ligand‐bound AR, the S791 residue is buried inside the protein core, suggesting that regulation of this phosphorylation site likely happens before the AR binds to its ligand. This was the possibility offered by computational analyses of the AR folding pathway that revealed the existence of complex folding kinetics, including a folding intermediate in which residue S791 is exposed to the solvent.^42^ Therefore, residue S791 appears as a regulatory site representing a crossroad between a nonphosphorylated, viable ligand‐binding folding pathway of AR and a phosphorylated, nonviable folding pathway leading to AR degradation.

## 6 PHARMACOLOGICAL VALUE OF TARGETING FOLDING INTERMEDIATES

Conventional rational drug discovery is primarily based on identifying small molecules that can hinder the biological activity of a target protein by binding to pockets in its native structure. Unfortunately, this is not a viable therapeutic strategy for many pathologies. In particular, neurodegenerative diseases rooted in protein misfolding and aggregation are paradigmatic examples of pathologies for which conventional therapeutic methods are largely unsuccessful.

The observation that a protein's physiological regulation can occur at the level of the folding pathway inspired us to test the possibility of pharmacologically modulating the expression of a given protein by targeting folding intermediates.^30^, ^42^, ^43^ Strategies to modulate protein folding kinetics using small peptides have been based on theoretical simulations and in‐vitro biophysical kinetic experiments. In particular, Das et al. employed a variety of biophysical experiments and coarse‐grained simulations to show that small peptides that provide a template secondary motif in the folding nucleus of a target protein can speed up its folding kinetics.^44^ Broglia et al. used a lattice‐based coarse‐grained model to theoretize the design of small peptides capable of docking to specific regions of a polypeptide and inhibiting its folding.^45^

**FIGURE 2:**
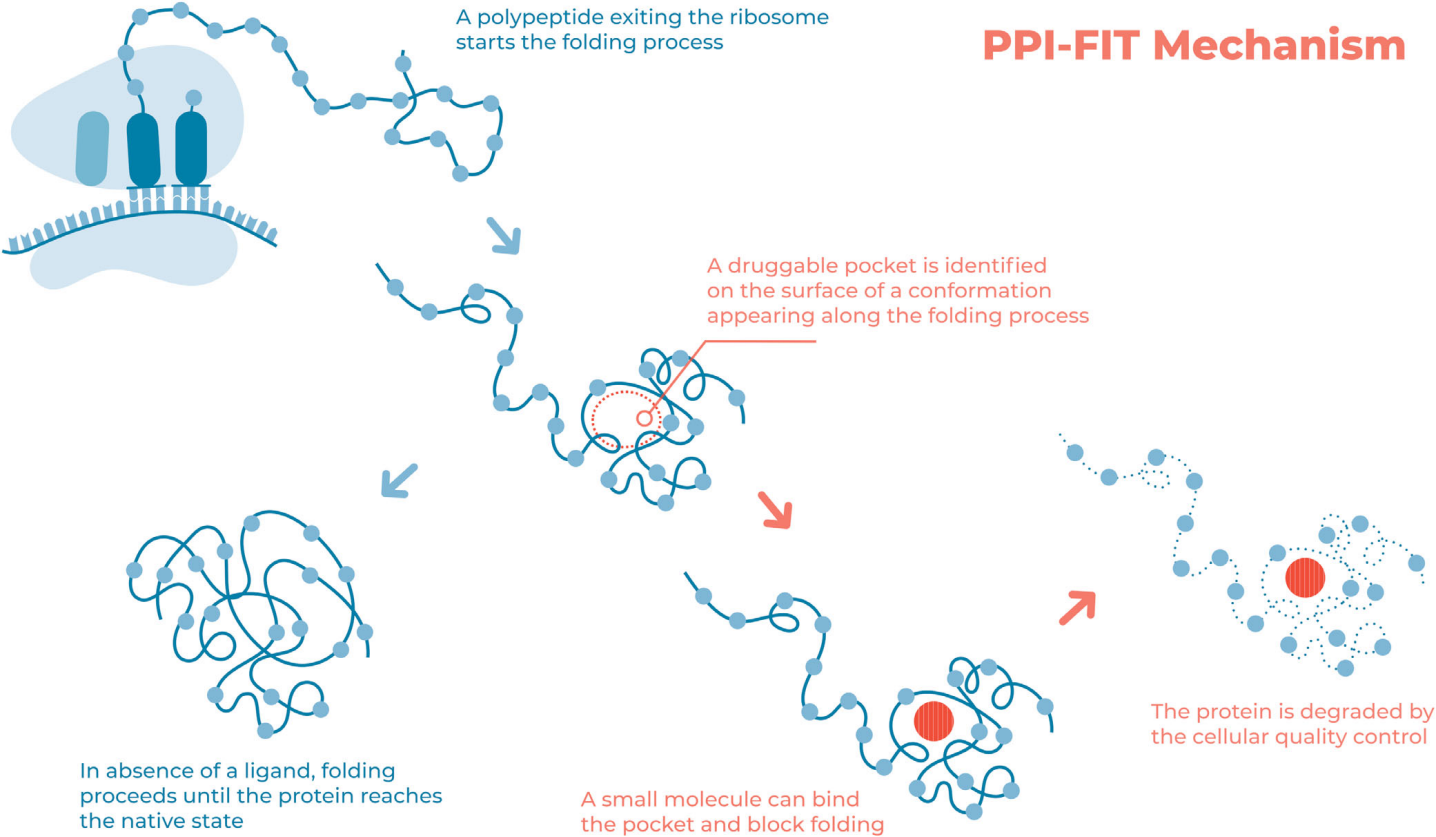
Rationale of targeting folding intermediates pharmacologically. The image illustrates the rationale underlying the pharmacological protein inactivation by folding intermediate targeting (PPI‐FIT) paradigm. Polypeptides exiting the ribosomal channel start the folding process. Folding intermediates transiently appearing along this process could be targeted with small molecules binding to unique pockets on their surface. Such a binding event ultimately interferes with the protein folding process, prompting the quality control machinery of the cells to reroute the polypeptide to degradation pathways.

The design of small molecules that suppress the expression of target proteins by acting on folding pathways was made possible by the development of a completely new paradigm for rational drug discovery named pharmacological protein inactivation by folding intermediate targeting (PPI‐FIT; Figure 2). In contrast to the conventional rational drug discovery approach, PPI‐FIT is based on reducing the cellular level of a target protein through small molecules that bind to specific folding intermediates, thus triggering protein degradation via the cell's protein folding quality control machinery. The rationale is that the cellular quality control machinery recognizes artificially stabilized intermediates as improperly folded species. In practice, the PPI‐FIT protocol is made possible by a novel generation of molecular simulations based on the enhanced sampling schemes discussed in Section 2, which enable the prediction of the folding process of the target protein with an atomic degree of resolution. Virtual screening campaigns are then run to identify small molecules that can bind to pockets in long‐lived protein configurations that are transiently visited only during the folding process and are not present in the native state. The PPI‐FIT can be applied in virtually all therapeutic areas and protein targets, including those considered undruggable with conventional methods.

Indeed, the first proof‐of‐concept application of PPI‐FIT led to the identification of small molecules capable of lowering the levels of the cellular prion protein in a dose‐dependent fashion, with no detected effect on a control protein with identical biogenesis. Biochemical assays performed with one of these small molecules (named SM875) demonstrated that the level of PrP in different cell types is reduced by promoting the lysosomal‐dependent degradation pathway. Further essays on inducible cell cultures showed that the molecule only acts on nascent proteins, while it shows no effects on native forms of the protein. Remarkably, SM875 also suppresses prion replication in cell cultures.^30^ The PPI‐FIT methodology is now being industrially exploited to identifying drug candidates in different therapeutic areas.^46^

## 7 CONCLUSIONS

The elucidation of the sequence of structural rearrangements through which globular proteins spontaneously reach their active native state (often referred to as the “Part II of the protein folding problem”) has been challenging theoretical and experimental biophysicists and biochemists for several decades. Until recently, such an intense research activity has mostly focused on addressing fundamental chemical‐physics questions: how can these polypeptide chains avoid the exhaustive exploration of their huge configuration space to efficiently and rapidly reach their native state? Is the folding mechanism sequential and deterministic, or is it completely heterogeneous and stochastic? What is the rate‐limiting step in folding, and what is the origin of the underlying free‐energy barrier? From a biological perspective, biological interest in understanding folding has mainly concerned pathogenic implications of misfolding. In contrast, the details of productive folding transitions have mostly been regarded as having little physiological relevance, assuming that the function and regulation of proteins only involved interactions with the polypeptide's native structures.

Over the last decade, however, several theoretical and experimental studies have challenged this view, suggesting that protein folding intermediates may be involved in many critical biological processes. In this perspective, A1AT serpin provides an extreme paradigmatic example of a biologically active protein in a metastable state on the folding pathway. Furthermore, in many cases, folding intermediates may be the only states where a polypeptide chain can undergo PTMs. In addition, many proteins are believed to be able to reach their stable state only upon binding to specific targets, suggesting the existence of complex feedback‐based regulatory mechanisms.

Reconstructing folding pathways at high resolution also provides an entirely new class of pharmacological targets, potentially paving the way for treating diseases for which conventional rational drug discovery schemes are inefficient or inapplicable. Examples involve proteins which do not display druggable pockets in their native structure or proteins responsible for misfolding diseases.

Several factors have driven this paradigmatic change of perspective on the role of protein folding intermediates. On the one hand, the development of sophisticated time‐resolved experimental techniques has revealed that the folding kinetics of biologically relevant proteins is far more complex than that of small globular domains, as it typically involves many time scales and rate‐limiting steps.^4^ On the other hand, the development of powerful algorithms and accurate models has made it possible to reconstruct these folding events at various degrees of resolution, ranging from relatively coarse‐grained to fully atomistic. While the initial seminal theoretical activity of the previous decades focused on so‐called native‐centric models that emphasize the role of the native structure in shaping the folding pathways, several models or approaches developed in the last 15 years have made it possible for the first time to predict folding pathways while retaining the information about the chemical composition of the chain. This progress paves the way to investigating the impact of mutations on folding kinetics and folding efficiency and the role of non‐native interactions in shaping the folding pathway. Finally, various methods are currently being developed that coherently integrate experimental information into numerical simulations, for example, Bayesian statistics approaches.^47^, ^48^ These schemes may correct for possible inaccuracies of the underlying atomistic models. At the same time, powerful machine learning and information‐theory‐based techniques are being exploited to obtain reliable multiscale and coarse‐grained models (for a recent review, see Reference 49) to consistently analyze vast amounts of data generated in simulations^50^ and to enhance sampling.^51^

In the next decade, the combination and integration of all these experimental and theoretical developments will likely have a great impact by generating a breakthrough in the appreciation of biological aspects connected with the folding mechanisms, potentially unveiling additional layers of complexity in the fundamental dogma of molecular biology.

## CONFLICT OF INTEREST

The authors are co‐founders and share holders of Sibylla Biotech SPA, a drug discovery company.
